# Clinical characteristics, treatment, and prognosis of pediatric Vasculo-Behçet’s syndrome: a retrospective analysis of 12 cases

**DOI:** 10.3389/fped.2026.1799746

**Published:** 2026-05-22

**Authors:** Ming Li, Xue Zhao, Lian Wang, Tong Yue, Min Wen, Fei-Fei Wu, Yu-Fan Wang, Dan Zhang, Ying-Jie Xu, Jia Zhu, Min Kang, Yu-Chun Yan, Feng-Qi Wu, Gai-Xiu Su, Jian-Ming Lai

**Affiliations:** 1 Department of Rheumatology and Immunology, Capital Center for Children’s Health, Capital Medical University, Capital Institute of Pediatrics, Beijing, China; 2 Department of Pediatrics, The Second Hospital of Hebei Medical University, Shijiazhuang, China; 3 Department of Pediatrics, General Hospital of Ningxia Medical University, Yinchuan, China; 4 Department of Radiology, Capital Center for Children’s Health, Capital Medical University, Capital Institute of Pediatrics, Beijing, China

**Keywords:** children, clinical characteristics, differential diagnosis, prognosis, vasculitis, Vasculo-Behçet's syndrome

## Abstract

**Objective:**

Vasculo-Behçet’s syndrome (VBS) is a rare subtype of Behçet's syndrome (BS) characterized by vascular involvement, and its clinical profile in the pediatric population remains poorly characterized due to limited available data. This study aimed to describe the clinical characteristics, treatment strategies, and prognosis of pediatric VBS and to provide evidence for early identification and clinical management.

**Methods:**

A retrospective analysis was performed on the clinical data of 12 pediatric patients (≤18 years old) with VBS admitted to three centers from January 2013 to December 2023. Demographic data, clinical manifestations, laboratory results, treatment regimens, and follow-up outcomes were collected and analyzed.

**Results:**

Among the 12 patients, there were 5 boys and 7 girls, with a median age at onset of 9.5 years (range: 3–13 years). Arterial involvement was observed in 10/12 cases, mainly characterized by vascular wall thickening (6/12 cases) and luminal stenosis (5/12 cases), involving the pulmonary artery, aorta, and multiple other sites. Venous involvement was found in 5/12 cases, predominantly wall thickening with thrombosis (3/12 cases). Multisystem involvement was common, including the skin (10/12 cases), the gastrointestinal tract (9/12 cases), and the urinary system (6/12 cases). Inflammatory markers (CRP/ESR) were elevated in 11/12 cases. All patients received glucocorticoid therapy; 11/12 cases received it combined with immunosuppressants, 9/12 cases with biological agents, and 4/12 cases underwent surgical treatment. With a median follow-up of 2 years (range: 4 months–5 years), 8/12 cases achieved stable remission, 2/12 cases (both complicated by aneurysms) had multiple relapses, 1/12 case died of sudden cardiac death, and 1/12 case showed no improvement.

**Conclusion:**

Pediatric VBS is a rare and heterogeneous condition with frequent arterial involvement in this cohort. Vascular wall thickening may aid in the early recognition of this condition, while aneurysms may be associated with poorer outcomes.

## 1 Introduction

Behçet's syndrome (BS) is a chronic, multisystem vasculitis of unknown etiology, characterized by recurrent orogenital ulcers, uveitis, and potential involvement of the blood vessels, nerves, and the gastrointestinal tract (1–3). Vasculo-Behçet's syndrome (VBS), a severe subtype of BS, is a major cause of morbidity and mortality due to its aggressive vascular involvement (4, 5). In adults, VBS accounts for 12.8%–40% of BS cases, predominantly affecting veins with lower extremity deep vein thrombosis as the most common manifestation (6–8). However, pediatric VBS is extremely rare, with the majority of the existing literature limited to case reports or small case series (9–13), leading to an unclear understanding of its clinical characteristics, vascular involvement patterns, and optimal treatment strategies.

Notably, the vascular involvement pattern of pediatric VBS may differ from that of adults. Previous small-scale studies suggested a higher proportion of arterial involvement in children (9), but the evidence is insufficient to guide clinical practice. Additionally, pediatric VBS often presents with multisystem involvement, making differential diagnosis from other primary vasculitides [e.g., Takayasu arteritis (TAK) and Kawasaki disease] challenging (11, 13). Given the lack of standardized diagnostic and therapeutic guidelines for pediatric VBS, this retrospective study summarized the clinical data from 12 cases to clarify the condition’s clinical features, treatment responses, and prognostic factors, aiming to provide valuable references for clinical practice.

## 2 Data and methods

### 2.1 Study participants

Pediatric patients with VBS (≤18 years old) diagnosed at the Capital Center for Children's Health, Capital Medical University (10 cases); the General Hospital of Ningxia Medical University (1 case); and the Second Hospital of Hebei Medical University (1 case) from January 2013 to December 2023 were enrolled. The inclusion criteria were as follows: (1) meeting the 2014 revised International Criteria for Behçet's Disease (1); (2) meeting the definition of VBS, namely BS plus vascular involvement, as confirmed by imaging (Doppler ultrasound, CT, or MRI), including venous thrombosis and/or arterial lesions (2); and (3) complete clinical and follow-up data. The exclusion criteria were as follows: (1) other primary vasculitides (e.g., Takayasu arteritis and Kawasaki disease); (2) vascular lesions caused by trauma, infection, or other secondary factors; and (3) refusal of participation by the patients or their legal guardians.

This study was approved by the Ethics Committee of the Capital Center for Children's Health at the Capital Medical University and the Capital Institute of Pediatrics (Approval No.: SHERLLM2023039). Due to the retrospective nature of this study, all clinical data were de-identified, and the ethics committee waived the requirement for informed consent in accordance with national legislation and institutional guidelines.

### 2.2 Data collection

Clinical data were extracted from electronic medical records, including (1) demographic characteristics (gender, age, and age at onset), (2) clinical manifestations (mucocutaneous symptoms, systemic symptoms, and multisystem involvement), (3) imaging findings (type, location, and characteristics of vascular lesions), (4) laboratory results (blood routine, inflammatory markers, immunological indicators, and infection screening), (5) treatment regimens (glucocorticoids, immunosuppressants, biological agents, and surgery), and (6) follow-up outcomes (remission, recurrence, complications, and survival status).

### 2.3 Imaging and laboratory assessments

This study adopted a targeted imaging strategy rather than routine vascular imaging screening for all pediatric patients with Behçet's syndrome due to radiation exposure risk in pediatric patients and clinical needs. Specific indications for vascular imaging included (1) clinical symptoms suggesting vascular involvement (e.g., recurrent fever, hypertension, asymmetric blood pressure in the limbs, limb ischemia, or weakened or absent pulses), (2) persistently elevated inflammatory markers [C-reactive protein (CRP)/erythrocyte sedimentation rate (ESR)] (8); (3) multisystem involvement (e.g., combined gastrointestinal or neurological symptoms) (8); and (4) neurological symptoms, such as headaches, nausea/vomiting indicating intracranial hypertension, or seizures (14). Patients meeting the above indications underwent Doppler ultrasound, CT, or MRI.

Doppler ultrasound was used for peripheral arteriovenous and abdominal deep vein lesions. Contrast-enhanced CT was performed for the thoracic/abdominal aorta and its branches. A non-contrast brain MRI was used for intracranial vascular lesions.

All imaging data were independently reviewed by two experienced radiologists in a double-blind manner, with discrepancies resolved by a third radiologist.

The Doppler ultrasound criteria for vascular lesions were as follows: (1) Vascular stenosis: a localized reduction in luminal diameter compared to adjacent normal segments or contralateral normal segments, combined with an elevated peak systolic velocity. The stenosis rate was calculated using the European Carotid Surgery Trial method, i.e., stenosis rate = {(D − R)/D} × 100% (D = normal reference diameter at the lesion site, R = residual lumen at the lesion site); (2) Aneurysm: local vascular diameter exceeding 1.5 times the adjacent normal vessel diameter; (3) Vascular occlusion: absence of blood flow signal within the vessel lumen, accompanied by decreased distal blood flow velocity; (4) Arteriovenous thrombosis: solid echoes or filling defects within the vessel lumen; and (5) Wall thickening: intima-media thickness (IMT) exceeding normal reference values or exceeding the IMT of adjacent normal vessels (15, 16).

The contrast-enhanced CT criteria for vascular lesions were as follows: (1) Luminal stenosis: the same as the ultrasound criteria, often accompanied by wall thickening in the acute phase, with irregular edges at the stenotic segment; (2) Aneurysm: the same as the ultrasound criteria; (3) Vascular occlusion: the same as the ultrasound criteria; (4) Arteriovenous thrombosis: the same as the ultrasound criteria; (5) Vascular wall thickening: normal vessel walls are thin and barely visible or show only a thin ring-like shadow on contrast-enhanced CT. Ring-like enhancement with irregular edges indicates wall thickening (15, 16).

Non-contrast brain MRI or MR venography (MRV) was primarily used to screen for intracranial venous sinus thrombosis. Typical imaging features include (1) loss of flow-void signal in venous sinuses, (2) high signal intensity on T1-weighted images (T1WI) in subacute thrombosis, and (3) absent or reduced blood flow signal in affected venous sinuses on MRV (17).

Regarding laboratory assessments, routine blood tests were performed, and CRP, ESR, serum ferritin, immunoglobulin (IgG, IgA), antinuclear antibody (ANA), and anticardiolipin antibody were detected. Infection screening included an Epstein–Barr (EB) virus nucleic acid test and a tuberculosis infection T-cell spot test (T-SPOT.TB).

### 2.4 Treatment and follow-up

Treatment decisions were individualized based on the type and severity of vascular involvement, following the treatment algorithm proposed by Emmi et al. (2) and the 2018 European Alliance of Associations for Rheumatology recommendations for Behçet's syndrome (18). Treatment selection criteria were as follows. For patients with venous thrombosis in typical sites (e.g., limb deep vein thrombosis), glucocorticoids combined with immunosuppressants (e.g., azathioprine, cyclosporine, or methotrexate) were used as first-line therapy. Anticoagulant therapy was not routinely administered unless complicated by intracranial venous sinus thrombosis, given that immunosuppressive therapy rather than anticoagulation is considered the cornerstone for controlling inflammatory thrombosis in BS (2, 18). In patients with extensive thrombosis involving large vessels (e.g., the vena cava) or Budd–Chiari syndrome, cyclophosphamide was preferred as the immunosuppressant of choice (2). For patients with arterial involvement (e.g., vascular wall thickening, stenosis, or aneurysm), cyclophosphamide combined with high-dose glucocorticoids was used as the primary regimen (2, 18). For refractory cases or those with recurrent disease activity despite conventional immunosuppressive therapy, anti-TNF-α agents (infliximab or adalimumab) were added (2, 18). For patients with aneurysms at high risk of rupture or with critical complications (e.g., severe valvular regurgitation or limb ischemia), surgical intervention (e.g., aneurysm resection and aortic valve repair) or endovascular treatment was performed, with pre-operative immunosuppressive therapy to reduce post-operative complications (2). Oral prednisone (1–2 mg/kg/day, maximum 60 mg/day) or intravenous methylprednisolone pulse therapy (10–30 mg/kg/day for 3 consecutive days, maximum 1,000 mg/day) was used in severe cases or in those with major organ involvement, followed by gradual tapering. Tumor necrosis factor antagonists [infliximab 5–10 mg/kg at weeks 0, 2, and 6, then every 8 weeks; or adalimumab 40 mg (weight ≥30 kg) or 20 mg every 2 weeks (weight <30 kg)] or interleukin-6 antagonists (tocilizumab) were used in refractory cases. Patients were followed up every 3–6 months via outpatient visits or telephone interviews.

Follow-up indicators included vascular lesion recurrence, new involvement sites, treatment-related complications, and survival status. The median follow-up duration was 2 years. Based on our center's experience, disease activity assessment criteria were defined as follows: stable remission was defined as the disappearance of clinical symptoms, normalization of inflammatory markers (CRP, ESR), and no progression of vascular lesions on imaging for ≥6 months; relapse was defined as the emergence of new clinical symptoms, re-elevation of inflammatory markers, or imaging evidence of new vascular lesions or progression of existing lesions; and no improvement was defined as persistent clinical symptoms, inflammatory markers, or vascular lesions without improvement.

### 2.5 Statistical analysis

This study is a descriptive analysis. Quantitative data are expressed as median (range). Categorical data are expressed as n/N.

## 3 Results

### 3.1 General clinical data

Among the 12 patients, 5 were boys, and 7 were girls, with a median age at onset of 9.5 years (range: 3–13 years). The median interval from onset to detection of vascular lesions was 8 months (range: 3 weeks–2 years and 6 months). Notably, vascular lesions were detected concurrently with BS diagnosis in 10/12 cases. During the same period, VBS accounted for 19.7% (12/61) of all pediatric BS cases across the three centers.

### 3.2 Clinical manifestations

Multisystem involvement was common (Table 1). The most frequent mucocutaneous manifestations were recurrent oral ulcers (11/12) and skin lesions (10/12). Gastrointestinal involvement (9/12) and fever (9/12) were also prominent systemic features. Neurological involvement occurred in 5/12 cases, including cerebral venous sinus thrombosis (2/12) and cerebral infarction (1/12). Details of all the clinical manifestations are presented in Table 1.

**Table 1:** Baseline characteristics and systemic manifestations.

Case No.	Sex	Age at onset	Age at diagnosis	Time to detection of vascular lesions	Initial presentation	Oral ulcers	Genital ulcers	Perianal ulcers	Skin manifestations	Ocular manifestations	Neurological involvement	Cardiovascular manifestations	Gastrointestinal involvement	Other manifestations
1	F	8y 4m	8y 6m	2m	Fever, rash	Yes	Yes	No	Erythema nodosum, panniculitis	Optic neuritis	Yes	Hypertension, asymmetric blood pressure	Yes	Lymphadenopathy
2	M	13y 3m	13y 5m	2m	Fever, abdominal pain	Yes	No	Yes	Erythematous papules of the lower extremities	Optic neuritis, scleritis	Yes	Hypertension, asymmetric blood pressure	Yes	Weight loss
3	F	9y 6m	9y 6m	2y 6m	Fever, diarrhea, cough	Yes	Yes	No	Congestive erythema	Hypertensive retinopathy	Yes	Hypertensive crisis, ventricular septal hypertrophy	Yes	Lymphadenopathy
4	M	12y 9m	14y 5m	1y 4m	Cystic acne	No	No	Yes	Cystic acne	None	Yes	Hypertension, asymmetric blood pressure	No	Bilateral knee pain
5	M	10y 3m	10y 4m	3m	Fever, rash	Yes	No	No	Erythematous papules of the lower extremities	None	No	None	No	None
6	M	5y	5y 20d	20d	Fever, oral ulcers, rash	Yes	No	No	Induration and hyperpigmentation of the lower extremities	None	No	None	Yes	Weight loss
7	F	3y	4y	1y	Oral ulcers	Yes	Yes	No	Bilateral lower extremity erythema nodosum	None	No	Severe aortic regurgitation, cardiomegaly	Yes	None
8	F	11y 6m	12y 4m	10m	Fever, abdominal pain, oral ulcers, vulvar ulcers	Yes	No	Yes	Folliculitis	None	No	None	Yes	Weight loss
9	F	9y 1m	10y 1m	1y	Oral ulcers, vulvar ulcers	Yes	No	Yes	None	None	No	None	Yes	Sterile pyuria
10	F	7y 2m	7y 8m	6m	Oral ulcers, perianal ulcers	Yes	Yes	Yes	Gluteal follicular rash	None	Yes	None	Yes	Arthralgia
11	F	11y	11y 4m	4m	Oral ulcers	Yes	No	No	Foot ischemic ulcer	Follicular conjunctivitis	No	None	Yes	Arthralgia, hematuria
12	M	Unknown	Unknown	1y	Oral ulcers	Yes	No	Yes	None	None	No	None	No	None

“Yes” indicates the presence of the corresponding manifestation, “No” indicates the absence, and “Unknown” indicates missing data; age units: “y” = years, “m” = months, “d” = days.

### 3.3 Vascular lesion characteristics

Vascular involvement patterns are summarized in Table 2. Arterial involvement predominated (10/12 cases), with 7/12 cases showing isolated arterial involvement and 3/12 cases showing combined arteriovenous involvement; isolated venous involvement was present in 2/12 cases.

**Table 2:** Details of Vascular Lesions, Treatment, and Prognosis.

Case No.	Vascular Lesions	Treatment Regimen	Prognosis
1	Thickening of the right renal artery, celiac trunk, and superior mesenteric artery walls; stenosis/thrombosis of corresponding arterial lumens; multiple intracranial venous thromboses	Thalidomide + IVMP + CTX + infliximab (IFX) → ADA + MMF	Stable
2	Left lower pulmonary artery aneurysm; cerebral venous sinus thrombosis; multiple deep venous wall thickening + renal vein thrombosis	IVMP + CTX + IFX → ADA + left lower lobectomy + azathioprine (AZA) → glucocorticoids + IFX + MMF	Stable vascular lesions, cataracts, and new neurological lesions
3	Thickening of the brachiocephalic trunk and multiple abdominal aortic branch walls; multiple arterial stenoses/occlusions; coronary artery involvement	Glucocorticoids + methotrexate (MTX) + thalidomide → CTX → MMF	Stable
4	Multiple arterial wall thickening, stenoses/occlusions + bilateral great saphenous vein wall thickening + thrombosis	Glucocorticoids + IFX + AZA → tocilizumab	Sudden cardiac death
5	Bilateral lower pulmonary artery thromboses	Glucocorticoids + thalidomide + MTX	No improvement
6	No significant arterial lesions; right brachial vein wall thickening + thrombosis	IVMP + CTX + IFX	Stable, cataracts
7	Severe aortic regurgitation with coronary artery disease; pulmonary hypertension	Glucocorticoids + thalidomide + IFX + CTX + aortic valvuloplasty	Stable, cataracts
8	Multiple arterial wall thickening, stenosis	IVMP + CTX + ADA	Stable
9	Thoracoabdominal aortic wall thickening	Glucocorticoids + thalidomide + ADA	Stable
10	Pulmonary artery trunk and branch thrombosis; aortic dissection + aortic right coronary sinus aneurysm	Bentall procedure + IVMP + thalidomide + CTX + ADA → AZA + ADA + glucocorticoids	Stable vascular lesions, new skin lesions, and gastrointestinal lesions
11	Thickening of left subclavian artery, superior mesenteric artery, and bilateral femoral artery walls; occlusion of the bilateral posterior tibial arteries; stenosis of the bilateral anterior tibial arteries	Oral aspirin + glucocorticoids + intravenous methylprednisolone pulse (IVMP) + cyclophosphamide (CTX) → mycophenolate mofetil (MMF)	Stable
12	No arterial wall thickening; thrombophlebitis of right small saphenous vein, left great saphenous vein, right cephalic vein, and right dorsal foot vein	Thalidomide + hydroxychloroquine (HCQ) + glucocorticoids + MMF + adalimumab (ADA)	Stable

IVMP, intravenous methylprednisolone pulse therapy; CTX, cyclophosphamide; HCQ, hydroxychloroquine; MTX, methotrexate; MMF, mycophenolate mofetil; AZA, azathioprine; ADA, adalimumab; IFX, infliximab.

The predominant arterial lesion types were vascular wall thickening (6/12) and luminal stenosis (5/12), with aneurysm and occlusion each occurring in 2/12 cases. Affected arterial sites included the pulmonary artery (3/12), the aorta and its branches (4/12), and the superior mesenteric artery (3/12). Venous lesions included limb venous wall thickening with thrombosis (3/12) and cerebral venous sinus thrombosis (2/12, one of which was combined with multi-site venous thrombosis). Full details of the vascular lesion distribution are provided in Table 2.

### 3.4 Laboratory results

Inflammatory markers were markedly elevated in the majority of the patients, with CRP and ESR elevated in 11/12 cases (median CRP 79.5 mg/L; median ESR 76 mm/h). Common hematologic abnormalities included leukocytosis (8/11 cases; median white blood cell count of 16.91 × 10^9^/L), anemia (7/10 cases; median hemoglobin of 104 g/L), and thrombocytosis (8/10 cases; median platelet count of 412.5 × 10^9^/L). Serum ferritin was elevated in 5/11 cases. Immunological testing revealed elevated IgG and IgA in 6/10 cases each; the direct antiglobulin test was positive in 3/12 cases, and the ANA or anticardiolipin antibody was positive in 2/12 cases. All cases tested negative for EB virus nucleic acid and T-SPOT.TB.

### 3.5 Treatment and prognosis

Treatment regimens are detailed in Table 2. All the patients received glucocorticoid therapy; 11/12 cases were combined with immunosuppressants, and 9/12 cases received biological agents. Surgical intervention was performed in 4/12 cases for critical complications. With a median follow-up of 2 years (range: 4 months–5 years), 8/12 cases achieved stable remission, 2/12 cases (both with aneurysms) had recurrent disease activity, 1/12 case died of sudden cardiac death, and 1/12 case showed no improvement. Treatment-related complications included cataracts (3/12 cases) and cerebral infarction caused by thrombosis shedding (1/12 case).

## 4 Discussion

VBS is a severe subtype of Behçet's syndrome characterized by vascular inflammation and injury. While adult patients have traditionally been reported to present predominantly with venous involvement (6–8), pediatric cases remain rarely described, and their patterns of vascular involvement are not yet well defined. In this retrospective analysis of 12 pediatric patients with VBS, arterial involvement was frequent (10/12 cases), including 7/12 cases with isolated arterial involvement. Arterial lesions mainly manifested as vascular wall thickening and luminal stenosis involving multiple sites, including the pulmonary artery and aorta. The observed arterial involvement rate (83.3%) was higher than the 40.3% reported by Hu et al. (9) in Chinese pediatric VBS. Multi-regional comparative data further suggest possible age- and region-related differences; Italian studies have reported comparable arterial and venous involvement in pediatric BS (13), whereas Turkish adult VBS studies have reported extrapulmonary arterial involvement ranging from 6.4% (6) to 51.6% (7), and a Chinese adult VBS study reported that 54.9% of patients had arterial lesions with 29.4% having isolated arterial involvement (8). Taken together, these findings suggest that pediatric VBS may present with a heterogeneous vascular involvement spectrum, in which arterial involvement is relatively frequent, although larger studies are needed to clarify age-related and regional differences.

Differential diagnosis of isolated arterial involvement in pediatric VBS is a clinical challenge. Among the 7/12 cases with isolated arterial involvement in this study, two had pulmonary artery thrombosis, and 5/12 cases had vascular manifestations (such as aortic branch wall thickening and stenosis) similar to those of TAK. However, all patients had Behçet's disease-specific manifestations, including oral ulcers and/or vulvar ulcers, perianal ulcers, skin lesions, and gastrointestinal involvement (cases 8, 9, and 11 had terminal ileal ulcers, with intestinal pathology showing neutrophil-predominant inflammatory cell infiltration with vasculitis, consistent with Behçet's disease pathological features). Takayasu arteritis rarely presents with mucosal involvement (19), and the pathological changes are mainly granulomatous vasculitis, which are key differential points. This finding is consistent with the conclusion reported by Akkuzu et al. (20–22) that arterial lesions in adult BS can mimic Takayasu arteritis. Japanese scholars (11) also reported a 17-year-old adolescent patient with VBS who presented with a fever of unknown origin as the initial symptom, with vascular ultrasound and CTA showing wall thickening in the aorta, carotid artery, and their branches (brachiocephalic trunk, left common carotid artery, and left subclavian artery); the patient was ultimately diagnosed with VBS based on clinical features. Cases 3 and 7 in this study had coronary artery dilatation, requiring differentiation from Kawasaki disease. According to the 2017 American Heart Association Kawasaki disease diagnostic criteria, Kawasaki disease mainly occurs in children under 5 years old, with fever accompanied by acute mucocutaneous changes as the core manifestation, a self-limited course, and coronary lesions that primarily resolve within 6–8 weeks (23). Case 3 had an age at onset >5 years, and although case 7 had an age at onset <5 years, both had recurrent oral and genital ulcers with gastrointestinal involvement during the disease course. Furthermore, case 3 had multiple vessel involvement, while case 7 had severe aortic valve regurgitation—manifestations that cannot be explained by Kawasaki disease alone. These studies suggest that, for children with vascular imaging similar to Takayasu arteritis or Kawasaki disease, a systematic evaluation of recurrent mucosal ulcers, intestinal involvement, and characteristic pathological changes is the core basis for establishing a VBS diagnosis, effectively preventing misdiagnosis and delayed treatment.

Vascular wall thickening was a common imaging finding in this study, being observed in 6 of 12 cases with arterial involvement and often accompanied by major organ involvement. A Korean study of 22 adult patients with VBS found that 18.2% had wall thickening of the aorta and its major branches (24). Kutluğ Ağaçkıran et al. (25) further proposed that pulmonary arterial wall thickening may be a marker of major organ involvement, especially vascular involvement, in BS. Taken together with the pediatric cases in our cohort, vascular wall thickening appears to be an important imaging feature of pediatric VBS. Even in the absence of typical lesions such as aneurysms, this manifestation may suggest ongoing vasculitis activity or major organ involvement and warrants consideration in clinical assessment.

Regarding venous involvement, among the 5/12 cases with venous involvement in this study, 3/12 cases presented with limb venous wall thickening with thrombosis, all of which were diagnosed by vascular ultrasound. Karadeniz et al. (26) found through magnetic resonance venography that patients with VBS had diffuse concentric wall thickening at multiple sites, including the inferior vena cava, iliac veins, and femoral veins; Sevik et al. (27) confirmed through vascular ultrasound that venous endothelial inflammation can lead to venous wall thickening in patients with BS and induce thrombosis tendency. This suggests that venous involvement in VBS is essentially a complete process of vasculitis affecting the venous wall, and that venous wall thickening may be an early imaging feature of venous vasculitis. Therefore, venous assessment in pediatric VBS should not only focus on thrombosis detection or intracranial venous thrombosis but also pay attention to manifestations of venous wall thickening, which helps to more accurately judge vasculitis activity and improve the disease assessment system.

In terms of treatment, all the patients in this study received glucocorticoids combined with immunosuppressants and/or biological agents, with eight cases achieving stable remission. However, two cases with aneurysms had recurrent disease activity, suggesting that aneurysm formation may be a poor prognostic factor. Zhou et al. (28) studied 69 adult patients with VBS with aneurysms and found that after a mean follow-up of 5.5 years, 21.7% of patients experienced relapse, 14.5% died from serious complications such as myocardial infarction and aneurysm rupture, and the 5-year overall survival rate was only 87.2%, significantly lower than that of patients with VBS without aneurysms. This suggests that aneurysms may be important predictors of mortality and serious complications in patients with VBS, requiring aggressive immunosuppressive therapy combined with necessary surgical or interventional treatment. Biological agents (especially tumor necrosis factor antagonists) showed a certain efficacy in our cohort, with 6/12 patients achieving disease control after combined use of biological agents. Hu et al. (29) studied six refractory pediatric patients with BS treated with anti-TNF therapy and found that the disease activity score (BDCAF) significantly decreased at 1 month, 6 months, and 1 year after treatment (*P*-values of 0.042, 0.027, and 0.026, respectively). All patients gradually reduced their glucocorticoid doses until discontinuation within 1 year; their immunosuppressive load score significantly decreased after 6 months of treatment, and no serious adverse events, such as severe infections, occurred, demonstrating good steroid-sparing effects and safety. Combined with our findings, biological agents may be a potential treatment option for pediatric VBS, especially showing good efficacy and safety in refractory cases, but larger prospective studies are still needed to verify their long-term effectiveness in the pediatric population.

This study has the following limitations. (1) The retrospective study design may have led to data loss. (2) The small sample size (12 cases) with referral cases may have led to selection bias, but considering the scarcity of previous related research, this study can still provide valuable clinical references. (3) Genetic testing was not performed, making it impossible to clarify the specific role of genetic factors, but the majority of the cases had good treatment outcomes, suggesting that genetic testing is not an essential tool for clinical management. (4) The use of a targeted imaging strategy rather than routine vascular screening for all patients may have potentially underestimated the true incidence of vascular involvement; however, patients without initial vascular imaging were closely monitored during follow-up, and additional imaging was promptly performed if any suspicious vascular symptoms or signs emerged, with no vascular events identified in these patients during the follow-up period. Future multicenter, large-sample prospective studies are needed to further verify the vascular involvement characteristics of pediatric VBS and explore the specific mechanisms of genetic and regional factors.

## 5 Conclusion

In summary, pediatric VBS is a rare and heterogeneous condition, with frequent arterial involvement in our cohort. Vascular wall thickening was a common imaging finding and may aid in the early recognition of the condition, while aneurysms may be associated with poorer outcomes and warrant closer monitoring. Comprehensive treatment strategies combining glucocorticoids, immunosuppressants, and biological agents may be considered in clinical practice, although the optimal treatment strategy still requires further investigation.

## Data Availability

The original contributions presented in the study are included in the article/Supplementary Material, further inquiries can be directed to the corresponding author.

## References

[1] Batu ED. Diagnostic/classification criteria in pediatric Behçet’s disease. Rheumatol Int. (2019) 39(1):37–46. 10.1007/s00296-018-4208-9 30430200

[2] Emmi G Bettiol A Silvestri E Di Scala G Becatti M Fiorillo C Vascular Behçet’s syndrome: an update. Intern Emerg Med. (2019) 14(5):645–52. 10.1007/s11739-018-1991-y 30499073

[3] Kurt T Aydn F Sezer M Tekgöz PN Tekin ZE Çelikel E Performance of diagnostic criteria in pediatric Behçet’s disease. Rheumatol Int. (2022) 42(1):127–32. 10.1007/s00296-020-04777-0 33449161

[4] Qian YL Quan RL Chen XX Lin YY Jing XL Gu Q Imaging characteristics and prognostic factors of Behcet’s disease with arterial involvement: a long-term follow-up study. Eur J Radiol. (2024) 170:111206. 10.1016/j.ejrad.2023.111206 37995514

[5] Chattopadhyay A Jain S Naidu GSRSNK Dhir V Chhabria B Acharya N Clinical presentation and treatment outcomes of arterial involvement in Behçet’s disease: a single-centre experience. Rheumatol Int. (2022) 42(1):115–20. 10.1007/s00296-021-05022-y 34661711

[6] Tascilar K Melikoglu M Ugurlu S Sut N Caglar E Yazici H.. Vascular involvement in Behçet’s syndrome: a retrospective analysis of associations and the time course. Rheumatology (Oxford). (2014) 53(11):2018–22. 10.1093/rheumatology/keu233 24907156

[7] Alpagut U Ugurlucan M Dayioglu E. Major arterial involvement and review of Behçet’s disease. Ann Vasc Surg. (2007) 21(2):232–9. 10.1016/j.avsg.2006.12.004 17349371

[8] Fei Y Li X Lin S Song X Wu Q Zhu Y Major vascular involvement in Behçet’s disease: a retrospective study of 796 patients. Clin Rheumatol. (2013) 32(6):845–52. 10.1007/s10067-013-2205-7 23443336

[9] Hu D She CH Bao HF Zou J Cai JF Ye JF Clinical heterogeneity and five phenotypes identified in pediatric Behçet’s syndrome: a cohort study from Shanghai Behçet’s syndrome database. World J Pediatr. (2024) 20(8):801–8. 10.1007/s12519-023-00785-9 38315355 PMC11402839

[10] Ben-David Y Gur M Ilivitzki A Khoury A Bentur L Butbul Aviel Y. Atypical cardiopulmonary manifestations in pediatric Behçet’s disease. Pediatr Pulmonol. (2020) 55(12):3407–13. 10.1002/ppul.25068 32915509

[11] Matsumoto H Yashiro-Furuya M Fujita Y Asano T Mori T Sato S Vascular Behcet’s disease preceded by fever of unknown origin: usefulness of ultrasonography for the detection of large-vessel vasculitis. Tohoku J Exp Med. (2021) 255(2):163–9. 10.1620/tjem.255.163 34707017

[12] Baş Ikizoğlu N Atağ E Ergenekon AP Yilmaz Yeğit C Gökdemir Y Erdem Eralp E An adolescent presented with hemoptysis: pulmonary artery aneurysm in pediatric Behçet’s disease. Arch Rheumatol. (2019) 35(2):283–6. 10.46497/ArchRheumatol.2020.7459 32851380 PMC7406172

[13] Mastrolia MV Bettiol A Marrani E Maccora I Taddei E Pagnini I Behçet syndrome in children and adults: discovering similarities and differences by a comparative study. Rheumatology (Oxford). (2023) 62(SI2):SI189–95. 10.1093/rheumatology/keac347 35703922

[14] Sorgun MH Rzayev S Kural MA Erdoğan S Yücesan C. Cerebral venous thrombosis in Behçet’s disease patients compared to other causes of cerebral venous thrombosis: a retrospective study. Arch Rheumatol. (2016) 31(3):248–53. 10.5606/ArchRheumatol.2016.5749 29900956 PMC5827850

[15] Nielsen BD Seitz L Schmidt WA. Update in imaging for large vessel vasculitis. Best Pract Res Clin Rheumatol. (2025) 39(3):102060. 10.1016/j.berh.2025.102060 40180819

[16] Moretti JB Michael R Gervais S Alchourron É Stein N Farhat Z Normal pediatric values of carotid artery intima-media thickness measured by B-mode ultrasound and radiofrequency echo tracking respecting the consensus: a systematic review. Eur Radiol. (2024) 34(1):654–61. 10.1007/s00330-023-09994-2 37542654

[17] Sadik JC Jianu DC Sadik R Purcell Y Novaes N Saragoussi E Imaging of cerebral venous thrombosis. Life (Basel). (2022) 12(8):1215. 10.3390/life12081215 36013394 PMC9410175

[18] Hatemi G Christensen R Bang D Bodaghi B Celik AF Fortune F 2018 Update of the EULAR recommendations for the management of Behçet’s syndrome. Ann Rheum Dis. (2018) 77(6):808–18. 10.1136/annrheumdis-2018-213225 29625968

[19] Hiller N Lieberman S Chajek-Shaul T Bar-Ziv J Shaham D. Thoracic manifestations of Behçet disease at CT. Radiographics. (2004) 24(3):801–8. 10.1148/rg.243035091 15143229

[20] Akkuzu G Özgür DS Karaalioğlu B Yalçın Mutlu M Yıldırım F Erdoğan M Behçet’s syndrome resembling Takayasu’s arteritis with the distribution of arterial involvement: a case report and literature review. Eur J Rheumatol. (2023) 10(2):62–6. 10.5152/eurjrheum.2023.22043 37470383 PMC10544313

[21] Okabe K Izumi Y Miyashita T Irino K Kawahara C Jiuchi Y An usual case of vasculo- and neuro-Behçet’s disease with MEFV mutations. Nihon Rinsho Meneki Gakkai Kaishi. (2014) 37(1):61–7. 10.2177/jsci.37.61 24598070

[22] Ocon AJ Mehta VR Peredo-Wende R. Coeliac artery dissection as a rare manifestation of Behçet’s disease. BMJ Case Rep. (2018) 2018:bcr2018226039. 10.1136/bcr-2018-226039 30115723 PMC6101312

[23] Brown J Cavanna A. Calculated decisions: Kawasaki disease diagnostic criteria. Pediatr Emerg Med Pract. (2020) 17(1):CD1–2. 32065518

[24] Lee S Kang S Eun Y Kim H Lee J Koh EM Clinical characteristics and radiographic outcomes of vascular Behçet’s disease involving the aorta or its major branches. Clin Rheumatol. (2022) 41(6):1769–77. 10.1007/s10067-021-06031-9 35128590

[25] Kutluğ Ağaçkıran S Sünbül M Doğan Z Kocakaya D Kayacı S Direskeneli H Pulmonary arterial wall thickness increased in Behçet’s disease patients with major organ involvement: is it a sign of severity? Rheumatology (Oxford). (2023) 62(3):1238–42. 10.1093/rheumatology/keac452 35944203

[26] Karadeniz H Ucar M Mammadov T Mirzayeva LS Guler AA Kardas RC Diffuse generalized venulitis as the primary pathology of Behçet’s disease: a comprehensive magnetic resonance venography study. Semin Arthritis Rheum. (2023) 62:152246. 10.1016/j.semarthrit.2023.152246 37573753

[27] Sevik G Ergelen R Aaçkÿran SK Direskeneli H Alibaz-Oner F. Intima-media thickness of common femoral vein is increased in Behçet’s disease. Clin Immunol. (2023) 250:109306. 10.1016/j.clim.2023.109306 37024022

[28] Zhou J Shi J Liu J Sun L Li L Li C The clinical features, risk factors, and outcome of aneurysmal lesions in Behcet’s disease. J Immunol Res. (2019) 2019:9198506. 10.1155/2019/9198506 31930152 PMC6942855

[29] Hu YC Yang YH Lin YT Wang LC Yu HH Lee JH Clinical manifestations and anti-TNF alpha therapy of juvenile Behçet’s disease in Taiwan. BMC Pediatr. (2019) 19(1):232. 10.1186/s12887-019-1613-5 31296171 PMC6621989

